# The Evolution of Bees: Insights from ‘Omic Studies

**DOI:** 10.1093/gbe/evaf226

**Published:** 2026-01-05

**Authors:** Dova Brenman-Suttner, Amro Zayed

**Affiliations:** Department of Biology, York University, Toronto, Ontario, Canada; Department of Biology, York University, Toronto, Ontario, Canada

**Keywords:** genomics, phylogenomics, regulatory RNAs, environmental ‘omics, evolution

## Abstract

Bees are important global pollinators that play a vital role in maintaining ecosystems and supporting global food production. They also exhibit a diversity of social organization, making them ideal model organisms for studying the evolution of sociality in animals. Recent advancements in genome sequencing have enabled researchers to address longstanding questions about the evolution of social behaviour in bees, particularly in the relatively few species that exhibit complex social structures, such as *Apis*. Whole genome phylogenies have enhanced our understanding of the complex evolutionary history of bees, providing a foundation for studying the evolution of specific traits, including eusociality. Recent transcriptomic and alternative splicing studies have advanced our understanding of how gene regulation and expression patterns contribute to behavioural plasticity, caste differentiation, and the emergence of social complexity. Comparative genomics across a range of bees with varying social behaviours has aided our understanding of the genomic features associated with social evolution and has shed light on its molecular underpinnings. Genomic approaches like GWAS and population genomic comparisons, combined with advanced sequencing technologies, have revolutionized the study of bee evolution, social behaviour, and environmental interactions. Pollen metabarcoding and environmental DNA (eDNA) techniques are now being used to quantify the intricate and complex interactions between bees and the plants they visit, and to identify other environmental factors, including pathogens that impact bee health. Additionally, techniques like museomics (using DNA from museum specimens) and broader genomic approaches have been instrumental in revealing how bees have been affected by anthropogenic changes. These tools offer valuable insights into population genetics, conservation biology, and the impact of environmental changes on bee populations. These advancements both provide critical insights into the molecular basis of eusociality and species adaptation and offer valuable tools for addressing the urgent challenges facing bee conservation due to anthropogenic change. By leveraging these genomic approaches, researchers can inform strategies for the preservation and sustainable management of bee populations worldwide.

Significance Bees are essential to global biodiversity and food security, making them key models for studying evolution, behaviour, and adaptation. Advances in genomics—spanning ultraconserved elements (UCEs), various environmental ‘omics approaches, and museomics—have revolutionized our understanding of bee phylogeny and social complexity. This review synthesizes recent findings on the molecular mechanisms driving the evolution of eusociality, a complex social system that has arisen independently multiple times. Regulatory processes including alternative splicing, long non-coding RNAs, microRNAs, and circular RNAs, shape phenotypic plasticity and social behaviour by modulating caste differentiation, reproductive roles, and task specialization. Together, these insights highlight the dynamic genomic architecture of social evolution and provide a foundation for future research in sociogenomics and adaptive evolution.

## Introduction

Bees are charismatic insects that are fundamentally important to humans on multiple levels. As pollinators, bees provide indispensable ecosystem services to a large community of flowering plants, thereby serving a critical role in maintaining plant biodiversity and that of the multitude of organisms that require flowering plants for survival and reproduction (Katumo et al. 2022). This of course includes humans as approximately one-third of global crop production, which represents species that are at least partially dependent on bee pollination, contributes to the human food supply (Khalifa et al. 2021). Some bees also produce honey, which has served as a human food staple since prehistoric times (Dunne et al. 2021), with the first evidence of managing honey bees coming from ancient Egyptian temples in 2450 BCE (Fraser 1931; Kritsky 2017). Beyond their ecological and economic importance, bees have also served as model organisms for the study of eusocial evolution, complex behaviour, and have even been used as field monitors for ecosystem quality and environmental change (Menzel 2012; Richards 2019; Wizenberg et al. 2025). Bees and their intriguing behaviours have also fascinated scientists for centuries. Darwin considered the “neuter” worker castes of bees a special difficulty for his theory of evolution by natural selection (Darwin 1809–1882), and the 1973 Nobel prize in Physiology and Medicine was partly awarded to Karl von Frisch for his work on deciphering the honey bee's infamous waggle dance (The Nobel Prize in Physiology or Medicine 1973). This fascination with bees and their biology was a large part of why the honey bee was selected as the third insect genome to be sequenced (after the fruit fly *Drosophila melanogaster* and the malaria vector *Anopheles gambiae*). Since the publication of the honey bee genome in 2006, numerous bee genomic resources have been developed. These include BeeBase (https://hymenoptera.elsiklab.missouri.edu/beebase), a comprehensive database for bee genomics that provides genome assemblies and annotations for various species, and the Hymenoptera Genome Database, which features HymenopteraMine (https://hymenoptera.elsiklab.missouri.edu), a data mining tool integrating genomic and biological information from numerous insect genomes. Additionally, the Sequence Read Archive (SRA) on the NCBI website offers extensive transcriptomic and genomic data for bees. To date, there have been 228 bee genomes sequenced (https://www.ncbi.nlm.nih.gov/datasets/genome/?taxon=34735) and 70 transcriptomes from 59 bee species (NCBI transcriptome shotgun assembly TSA; https://www.ncbi.nlm.nih.gov/Traces/wgs/).

Here we provide a review on the ‘omics tools and techniques that have shaped our understanding of bee biology and evolution. Taxonomy and phylogenetics are critical for understanding both patterns of biodiversity and phenotypic evolution. Advances in genomics have been instrumental in enhancing our understanding of the bee “tree of life”. For example, the availability of genome sequences has allowed researchers to identify ultraconserved elements that have proven very effective in generating high-quality phylogenies for a large group of bees. Understanding the genetics and molecular biology underlying social behaviour has been a goal of sociogenomics since its inception with the sequencing of the honey bee genome in 2006 (The Honeybee Genome Sequencing Consortium 2006). While we are still searching of the “genes for altruism” (Toth and Zayed 2021), research over the past decade has generated a great deal of knowledge on the emerging roles of alternative splicing and noncoding RNAs in generating behavioural plasticity in bees (Harpur et al. 2019; Shell et al. 2021; Traniello et al. 2023; Brenman-Suttner and Zayed 2024), which is an emerging topic that we also review here. Large genome datasets have also enabled cross-species comparisons that shed light on the evolution of social behaviour, allowing researchers to address some long-standing sociobiological questions. Finally, metagenomics and metabarcoding have opened the doors for fully exploring the plethora of interactions between bees and plants, leading to vast new insights into bee biology and health. Additionally, advances in sequencing preserved specimens have enabled researchers to “resurrect” bee genomes to understand threats to their population viability over time.

## Tools for Studying the Evolution of Bees

### Genomic Tools for Phylogenetic Analysis

With over 20,000 species belonging to 500 genera and seven families, bees are extraordinarily diverse in terms of morphology and behaviour (Almeida et al. 2023). Bees arose during the mid-Cretaceous period around 140 to 110 Mya (million years ago) along with flowering plants (Almeida et al. 2023; Ascher and Pickering 2024). The largest bee family, Apidae, includes 5,600 species spread across over 30 tribes and 170 genera, displaying a wide range of social behaviours (Michener 2007; Cardinal et al. 2010). The family Apidae includes several subfamilies that display a wide range of social behaviours. For example, the subfamily Apinae encompasses species with varying levels of social complexity: advanced eusocial species like honey bees (*Apis* spp.), primitively eusocial species such as bumblebees (*Bombus* spp.), and other eusocial lineages like stingless bees (*Meliponini*). It also includes orchid bees (*Euglossini*), which are largely solitary, as well as Nomadinae and Xylocopinae that exhibit a range of social behaviours (Michener 1974). Reconstructing the phylogenetic relationships among bee groups helps us understand when these diverse behaviours evolved. Phylogenetic studies are also valuable for understanding whether simpler forms of social behaviour, such as communal living, are prerequisites for the evolution of more complex eusociality, and to what extent reversals to solitary living occur (Romiguier et al. 2022). Phylogenomic analyses, which use entire genomes, offer deeper insights into evolutionary relationships compared to traditional molecular methods that rely on limited molecular data (Ribeiro and Espindola 2024). Advances in next-generation sequencing (Ballare et al. 2019; Johnson 2019) have allowed researchers to assemble and resequence the genomes of diverse bee species from across the evolutionary tree of life for phylogenetic reconstruction (Almeida et al. 2023; Henriquez-Piskulich et al. 2024). To date, 140 bee species belonging to 47 genera have been sequenced (https://www.ncbi.nlm.nih.gov/datasets/genome/), representing only 0.7% of the described bee species and 9.4% of the described genera. This limited genomic representation underscores the need for continued sequencing efforts, particularly in underrepresented lineages. Earlier phylogenetic studies, prior to the use of UCE-based methods, relied on multi-gene datasets. For example, Hedtke et al. (2013) combined datasets from 349 bee genera and analyzed 17,000 sites from nuclear protein-coding genes, confirming the monophyly of each bee family. It also supported Melittidae as the sister group to all other bee families (Hedtke et al. 2013), consistent with findings from Brady et al. (2011), but differing from other studies that place Melittidae as sister only to long-tongued bees (Danforth et al. 2006; Michez et al. 2009; Debevec et al. 2012; Cardinal and Danforth 2013). The integration of next-generation whole genome sequencing, ultraconserved elements (UCEs) (Gueuning et al. 2020), and DNA barcoding (Trunz et al. 2016; Praz et al. 2019) has significantly improved phylogenomic resolution, clarifying uncertain evolutionary relationships and challenging established phylogenies. In the following sections, we review major studies that have employed new techniques for generating data and their contributions to understanding the phylogeny of Apidae.

### Phylogenomics and Ultra-conserved Elements (UCEs)

**Fig. 1.**
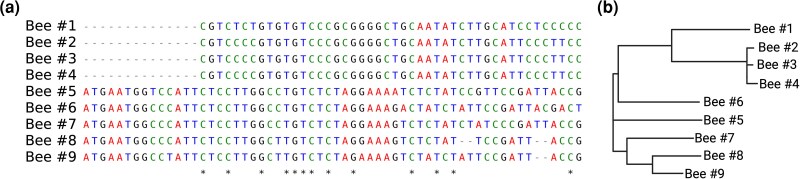
Ultraconserved elements (UCEs) are regions of the genome that are highly conserved across diverse species. Core UCEs are first identified by comparing reference genomes of two or more species to find stretches of conserved DNA. Next, many short bait sequences are generated that span the UCEs. These baits are used to capture matching DNA from species of interest. After hybridization with bait sequences, the DNA fragments that contain the UCEs plus the flanking regions are pulled out, cleaned and sequenced. Following filtering and alignment, genetic variation within UCE sequences can be used for phylogenetic inference. a) An example of a UCE alignment adapted from Faircloth et al. (2015). The asterisks identify conserved bases in this region. b) A hypothetical phylogenetic tree generated from the UCE shown in A.

Early phylogenetic relationships have traditionally been inferred using morphological traits, but these can be limited by low variation between taxa, polymorphisms within species, or geographic variation that is unrelated to speciation. DNA barcoding is one method of identifying phylogenetic relationships and is often performed using the cytochrome oxidase subunit 1 (COI) gene because it differs enough between species to allow for species-level resolution (Kowalska et al. 2019). The COI gene in combination with morphology has been used previously for bee identification (Pauly et al. 2019; Praz et al. 2019). However, COI can be unreliable due to mitochondrial inheritance issues and biases from mitochondria-specific evolutionary forces (Rubinoff et al. 2006; Desalle et al. 2017). Nuclear markers like EF-1α, 28S, and ribosomal ITS regions have also been used as a DNA barcoding method (Elbogen 2012; Arstingstall et al. 2021), but these can lack phylogenetic resolution or show within-genome variation, especially in insects (Rubinoff et al. 2006; Dietz et al. 2022). Because of these limitations, researchers have increasingly turned to genome-scale approaches. Among these, ultraconserved elements (UCEs) have emerged as a particularly powerful and cost-effective tool, especially for non-model taxa (Fig. 1). Despite their powerful resolution, these genomic tools are still limited by the lack of reference genomes overall, with many genera remaining unsequenced, making it difficult to build comprehensive phylogenies.

Ultraconserved elements (UCEs) are short genomic regions, usually larger than 200 bp (Cummins et al. 2024), that are highly conserved among taxa. While most UCEs are found in non-coding regions, some have been shown to overlap with protein-coding regions, as seen in humans (Bejerano et al. 2004; Snetkova et al. 2022). These regions have few repeats and, when captured along with adjacent DNA, can be used for phylogenetic reconstruction. Tree-building algorithms use UCE data to reconstruct evolutionary histories, accounting for the fact that DNA variability increases with distance from the UCE (Zhang et al. 2019). This approach is relatively inexpensive, enabling researchers to sequence over 1,000 target sequences containing exons and some intronic elements for analysis (Van Dam et al. 2021). Regions of high GC content can complicate phylogenetic reconstruction because they are often affected by GC-biased gene conversion (gBGC) following recombination events (Beye et al. 2006; Kent et al. 2012; Wallberg et al. 2015; Bossert et al. 2017). Although the average genomic GC content in honey bees is relatively low (∼33%) (Wallberg et al. 2019), in highly eusocial species like honey bees, which exhibit very high recombination rates, this process may increase GC content in specific regions of the genome even if the overall GC content remains low. These local increases in GC can influence substitution patterns and can complicate the generation of congruent gene trees, especially when gene divergence rates vary across species, a phenomenon known as incomplete lineage sorting (Freitas et al. 2021). To overcome these issues, researchers have increasingly been focusing on UCEs. UCEs are enriched in AT-content, which makes them less susceptible to the effects of gBGC in GC-rich regions. As a result, UCEs provide more consistent substitution patterns across taxa and improve the accuracy and congruence of both gene and species trees (Bossert et al. 2017).

UCEs have been widely applied to construct phylogenies at many taxonomic levels of the bee tree of life and have led to adjustments in the phylogenetic organization and divergence times for many of the seven families within Anthophila. For example, UCEs have uncovered an increase in diversification rates among Andrenidae over the past 15 million years, particularly within the genera *Andrena* and *Perdita*, representing some of the fastest diversifying bees (Bossert et al. 2022 ). UCEs have also helped refine the phylogeny of the Halictidae, the second-largest bee family, by revealing that the Middle Eastern *Clavinomia* is closely related to the North American *Dieunomia*, leading to the inclusion of *C. clavicornis* in the tribe Dieunomiini (Bossert et al. 2024). Additionally, UCEs confirmed that *Homalictus* is a subgenus within *Lasioglossum*, while reinstating *Rostrohalictus* as a subgenus for *Lasioglossum longirostre* (Zhang et al. 2022). In Megachilidae, UCEs have helped date the origin of the mason bee subgenus *Osmia* to the late Miocene in the West Nearctic, around 5.5 to 4.8 mya, before its dispersal from the East Palearctic (Branstetter et al. 2021). Collectively, these studies highlight the role of UCEs in establishing accurate timelines and taxonomic classifications that were previously difficult to determine with confidence, especially among non-model organisms.

Phylogenomic studies using UCEs have greatly advanced our understanding of bee evolution within the Apidae, which exhibit diverse ecological and behavioural traits (Orr et al. 2022). One of the most influential studies reorganized the subfamilial relationships within Apidae (Bossert et al. 2019). This study confirmed the monophyly of Centridini and showed that they belong with the corbiculate bees among the Apinae. It also clarified the phylogenetic positions of the oil-collecting genera *Ctenoplectra* and *Tetrapedia*, showing them as sister groups and belonging to the Xylocopinae, and suggesting shared life history traits such as large egg sizes and co-evolutionary patterns with phoretic mites (Bossert et al. 2019). These insights shed light on the evolution of specialized pollen-collecting structures. The study also resolved the long-standing debate over cleptoparasitism in Euglossini, supporting a single origin of the trait rather than two independent occurrences (Bossert et al. 2019).

UCE-based studies have made significant contributions to understanding the subfamily Eucerinae, which includes the long-horned bees—a difficult group to categorize due to their similar external morphology (Freitas et al. 2023). For instance, an early study using UCEs identified three main clades within Eucerinae (Freitas et al. 2021), while a follow-up study expanded this to seven clades and identified four new subtribes: Alloscirteticina, Gaesischiina, Thygaterina, and Melissodina (Freitas et al. 2023). The latter study also estimated that the diversification of Eucerini began around 20 million years ago, suggesting the group likely originated in southern South America and the Andean region before dispersing northward (Freitas et al. 2023). Similarly, UCEs have played a key role in clarifying the origins of brood parasitism within the cleptoparasitic subfamily Nomadinae. A study using UCEs across 114 species confirmed the monophyly of the Nomadinae, revealing that all tribes within the subfamily descend from a single parasitic ancestor (Sless et al. 2022). This research demonstrated that parasitism initially evolved within hosts from the same family, Apidae, before some lineages shifted to parasitizing Andrenidae, among other families (Sless et al. 2022). Another study using 2,545 UCEs provided the first global molecular phylogeny of the genus *Nomada*, confirming its monophyly and dating its divergence from other Nomadinae to the late Cretaceous period in the Nearctic (Odanaka et al. 2022). Overall, UCE-based phylogenomic studies have revolutionized our understanding of bee evolution within Apidae. By providing a consistent framework for classification and resolving longstanding taxonomic challenges, these studies have uncovered the complex evolutionary history and ecological dynamics of bee subfamilies.

Understanding the phylogeny of tribes within the subfamily Apinae is important as it has implications for understanding the evolution of eusocial behaviour. Several previous studies based on morphology, behaviour, or earlier molecular approaches suggested that Euglossini diverged first within Apinae, followed by the primitively eusocial Bombini with the advanced eusocial Meliponini and Apini as sister groups where eusociality elaborated, thus suggesting a single origin of eusociality and a single origin of advanced eusociality in this subfamily (Cameron 1993; Mardulyn and Cameron 1999; Cardinal and Packer 2007; Romiguier et al. 2016). However, a UCE-based study provided evidence for a different topology within the Apinae subfamily that has been supported by previous morphological and behavioural studies (Woodard et al. 2011; Hedtke et al. 2013), which proposes Euglossini as the basal group, followed by Meliponini and Bombini as sisters to Apini (Bossert et al. 2017). By rearranging the placement of these groups, we now must consider that there are dual origins of advanced eusociality within corbiculate bees.

## Alternative Splicing, Regulatory RNAs and the Molecular Basis of Eusocial Evolution

**Fig. 2.**
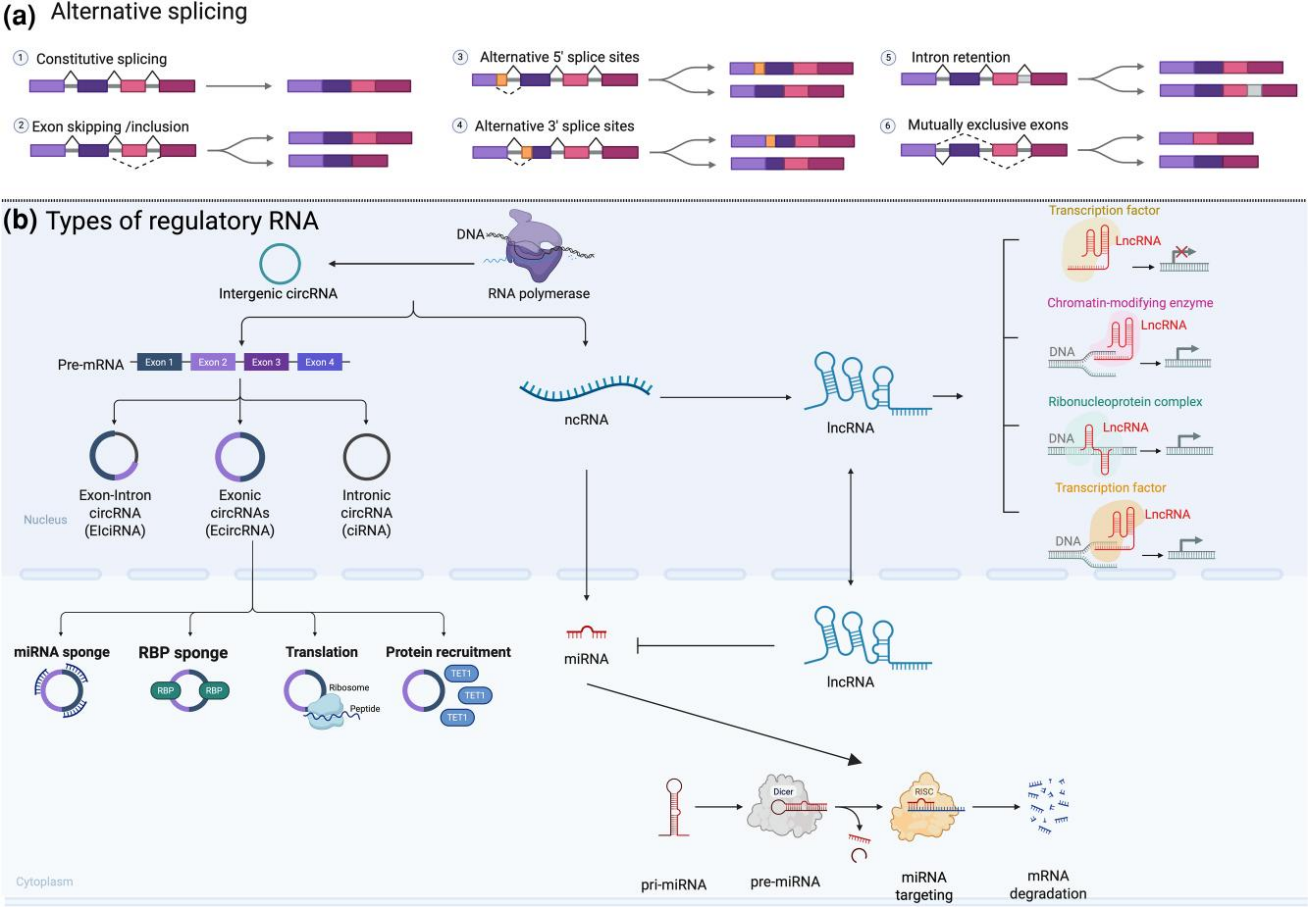
Overview of alternative splicing mechanisms and types of regulatory RNAs that underpin eusocial behavior in bees. a) Alternative splicing generates transcript diversity through various mechanisms, including constitutive splicing, exon skipping/inclusion, alternative 5′ or 3′ splice sites, intron retention, and mutually exclusive exons. These events result in different mRNA isoforms from the same gene. b) Regulatory non-coding RNAs (ncRNAs), including circular RNAs (circRNAs), microRNAs (miRNAs), and long non-coding RNAs (lncRNAs), are transcribed from pre-mRNA or intergenic regions. CircRNAs can act as miRNA sponges, RNA-binding protein (RBP) sponges, or participate in translation and protein recruitment. LncRNAs modulate gene expression through interactions with chromatin, ribonucleoprotein complexes, or transcription factors, or by inhibiting microRNAs. miRNAs are processed from non-coding RNAs and are involved regulating gene expression by binding to target mRNAs in the RISC complex that are then degraded. Each of these mechanisms has been implicated in either regulating bee behaviour or the evolution of bee behaviour.

Eusociality, characterized by overlapping generations, reproductive division of labour and cooperative brood care, is a major evolutionary transition that has independently evolved multiple times in bees (Wilson 1971). Understanding the evolutionary and mechanistic perspectives of this transition remains a central focus of sociobiology, where many recent studies have focused on identifying the regulatory mechanisms underlying this behaviour. For example, epigenetic mechanisms, which are stable alterations to gene expression that do not change the DNA itself that arise during development or due to environmental changes, are known to be important for eusocial evolution (Jaenisch and Bird 2003). These mechanisms play an important role in regulating gene expression, thus facilitating behavioural adaptations essential for social evolution, as existing genes can be reversibly modified to adjust behaviour (Kribelbauer et al. 2020). Much of the work on epigenetics in social insects has implicated DNA methylation (Herb 2014) and acetylation (Spannhoff et al. 2011) as important for social behaviours, including regulating the division of labour (Lyko et al. 2010; Foret et al. 2012; Araujo and Arias 2021), and maternal care (Rehan et al. 2016; Arsenault et al. 2018). However, confirming these findings has been challenging, often yielding contradictory results (Kapheim et al. 2015; Libbrecht and Keller 2015; Standage et al. 2016). With the advancement of genome sequencing technologies, it has become easier to confidently identify the role that other genomic and transcriptomic components play in regulating behaviour, such as alternative splicing and non-coding RNA (ncRNA), which include long non-coding RNA (lncRNA), microRNA (miRNA), and circular RNA (circRNA; Fig. 2). In the following sections, we outline the contribution of genomic studies in identifying RNA or regulatory elements that have been implicated in social phenotypes or social evolution of bees. We focus on studies that incorporate a diverse set of bees displaying varying levels of social complexity that help uncover the underlying patterns linked to social behaviour at the genomic level.

### Alternative Gene Isoforms

Alternative splicing, a conserved, posttranscriptional regulatory process, is important for generating phenotypic diversity by producing different isoforms (Kannan et al. 2019; Wright et al. 2022). This flexibility provides raw material for natural selection to act upon, contributing to the evolutionary diversification of traits, especially in taxa like bees that have evolved to have complex social structures. Alternative splicing involves several mechanisms, including exon skipping, alternative 5′ or 3′ splice sites, which alter exon length by splicing before or after the exon at the 5′ or 3′ end; intron retention, where the intron is not spliced out; and alternative first or last exons, where the first or final exon of a transcript is either retained or spliced out (Wright et al. 2022). By employing these mechanisms in different combinations, a diverse array of mRNA products can emerge from a single genomic template, thereby introducing numerous avenues for phenotypic variation (Bush et al. 2017). Early investigations into alternative splicing in honey bees were pivotal in elucidating its role in sex determination among bees. In honey bees, the splicing cascade is triggered by the complementary sex determiner (CSD) which is a mechanism by which heterozygosity at the *csd* locus will lead to development of females, and hemizygosity or homozygosity will result in males (Otte et al. 2023). One of the important genes in this pathway is the feminizer gene (*fem*), which influences the sex-specific splicing of doublesex (*dsx)* and consequently affects male or female development (Gempe et al. 2009). One recent transcriptomic study highlighted the importance of alternative splicing in sex determination and found that embryos at various developmental stages had an increasing number of alternatively spliced genes specific to sex as development progressed (Netschitailo et al. 2022). Patterns of alternative splicing have been found to greatly differ between castes in *Apis mellifera*, with more isoforms produced among the worker phenotype (Zheng et al. 2023). These caste-specific splicing patterns likely play a role in the evolution of eusociality by allowing distinct behavioural and physiological roles to emerge from a shared genetic background. While alternative splicing provides a flexible means of generating transcriptomic diversity from a static genome, it operates alongside epigenetic mechanisms such as DNA methylation and histone modification, which also play a significant role in shaping caste-specific gene expression (Kucharski et al. 2008; Foret et al. 2012; Wojciechowski et al. 2018). These epigenetic modifications can influence splicing decisions as well, creating a complex regulatory network that integrates multiple layers of gene regulation to produce the distinct phenotypes observed in eusocial bees. Follow-up studies have shown that many alternatively spliced genes are often involved in processes important for social behaviour, such as communication (Liu et al. 2024). Similarly, in *Bombus terrestris* colonies, alternative splicing has been implicated in differences in reproductive workers and queens versus non-reproductive workers and males with around 40% of the expressed genes exhibiting multiple isoforms, some of which have been linked to genes involved in the ecdysteroid pathway, which influences various behaviours (Price et al. 2018). This suggests that alternative gene splicing has been co-opted independently across bee lineages to facilitate reproductive division of labour, which is essential for more advanced forms of sociality in bees. Alternative splicing also has implications for foraging behaviour, as seen in the *elavl2* gene, an ortholog of the conserved ELAV/Hu family of RNA-binding protein genes (Samson 2008; Ustaoglu et al. 2021). This gene can produce 40 isoforms, two of which are involved in learning and memory consolidation, which are crucial components of foraging behaviour in worker honey bees (Ustaoglu et al. 2021). Overall, alternative splicing appears to be an important process for generating phenotypic plasticity, especially in social insects where different castes often arise from the same genome. This plasticity not only underpins current social organization but likely contributed to major evolutionary transitions in social complexity across bee lineages. The diverse mechanisms of alternative splicing produce a wide array of mRNA isoforms, revealing complex regulatory networks that underpin social insect biology. Future research should aim to uncover further connections between alternative splicing and social behaviours, such as identifying behaviour-specific or caste-specific isoforms or uncovering regulatory isoforms that influence key aspects of social behaviour.

### Long Non-Coding RNA

Long non-coding RNAs (lncRNA) are a class of non-coding RNA transcripts exceeding 200 nucleotides long that lack protein-coding capacity (Kapranov et al. 2007). They also play diverse roles in gene regulation through their ability to alter chromatin structure, which affects both local and distant transcription (Statello et al. 2021). They are transcribed from promoters, enhancers, or intergenic regions (Fang and Fullwood 2016) and have a 5′ cap (Tamtaji et al. 2021) and polyadenylated tail (Carninci et al. 2005). These transcripts can localize to both the nucleus (Derrien et al. 2012) and cytoplasm (Rashid et al. 2016), and are involved in mRNA stability, translation regulation, and microRNA (miRNA) biogenesis, or interference in a tissue-specific manner (Ingolia et al. 2011; Jayakodi et al. 2015; Montes et al. 2015; Paraskevopoulou and Hatzigeorgiou 2016; Noh et al. 2018). In bees, over 21,000 lncRNAs have been identified (Zheng et al. 2023), and several studies have highlighted their connection to various developmental processes and social behaviours. These findings suggest that lncRNAs may have contributed to the evolution of eusociality in bees, enabling fine-tuning regulation of traits that are essential for caste differentiation and task specialization. For instance, lncRNAs such as *lncov1* and *lncov2* and others map to a QTL that has been previously linked to ovary size (Humann et al. 2013) and oviposition (Chen et al. 2017), brain function in drones (Sawata et al. 2002), communication through the waggle dance (Feng et al. 2022), and are linked to the transition from nurse to forager roles by modulating hormone levels (Amdam et al. 2003; Drapeau et al. 2006; Tadano et al. 2009; Liu et al. 2019). A specific lncRNA, *kakusei* in honey bees, has been associated with multiple behaviours and processes important for sociality, including foraging behaviour (Singh et al. 2020) and learning and memory (Kiya et al. 2007). Its homolog, *Acks*, was found to be involved in temperature sensing and thus important for hot defensive bee ball activity in fighting giant hornets (Kiya et al. 2012).

Several studies have associated various lncRNAs with immune responses in different honey bee species and subspecies, aiding our understanding of the consequences of disease transmission within a social colony. For example, long intergenic non-coding RNAs (lincRNAs) were found to be upregulated in bees infected with deformed wing virus (DWV) or Sacbrood virus (SBV) in *Apis cerana* (Jayakodi et al. 2015). Additionally, differentially expressed lncRNAs (DElncRNAs) were identified in both *Apis cerana* (Guo et al. 2023) and *Apis mellifera ligustica* (Ye et al. 2022) following infection with the chalkbrood disease-causing fungal pathogen *Ascosphaera apis*, or following *Nosema ceranae* infection in *Apis cerana* (Wang et al. 2023). The lncRNAs identified in these studies were involved in the regulation of immune-related gene expression pathways, suggesting their role in the defense mechanisms of social bee colonies (Guo et al. 2023; Wang et al. 2023). Furthermore, exposure to insecticides like the neonicotinoid Dinotefuran can induce lncRNA responses, further highlighting their involvement in the response to environmental stressors through immune system activation (Huang et al. 2021). Overall, lncRNAs in bees serve multifaceted roles in development, behaviour, and immune function, shedding light on the intricate regulatory mechanisms within social insect colonies. Their involvement in caste-related traits, behavioural transitions, and immune responses suggests they may have played an important role in the evolutionary origins and elaboration of eusociality across bee lineages.

### MicroRNA

MicroRNAs, short sequences of 18 to 24 nucleotides, are integral components of the gene silencing program within the RNA-induced silencing complex (RISC), enabling precise and targeted silencing of mRNA (Sovik et al. 2015; Bartel 2018). The advancement of genome sequencing technologies has led to the development of miRbase (www.mirbase.org), a comprehensive database encompassing microRNA profiles and annotations across 271 organisms, facilitating comparative analyses to identify both conserved and novel microRNAs (Kozomara et al. 2019). Early studies have highlighted the importance of microRNAs in regulating the timing and spatial expression of specific genes during development, suggesting their role in constraining the plasticity of certain genes by regulating their expression patterns (Peterson et al. 2009). This precise regulatory control may have been instrumental in enabling caste differentiation from a shared genome, supporting the evolution of eusociality in bees. However, the extent to which this regulation directly drives specific developmental outcomes remains an area of ongoing investigation, as many of the studies are correlational. Recent studies have revealed that, while microRNAs themselves might not be the primary drivers of eusocial genome evolution, their coevolution with target sequences significantly influences various social behaviours (Kapheim et al. 2020b). In a study by Vieira et al. (2021), two microRNAs, *miR-34* and *miR-317*, were found to be upregulated in queens relative to workers. These microRNAs downregulate the gene *Takeout*, which leads to a reduction in juvenile hormone levels. This hormonal shift is thought to promote neurogenesis, the process by which new neurons are formed in the brain (Vieira et al. 2021).

MicroRNAs are also suggested to be involved in caste-specific development processes. Distinct expression patterns of microRNAs have been observed in queen and worker pupae (Weaver et al. 2007) and larvae (Shi et al. 2015; Ashby et al. 2016) of *Apis mellifera*, as well as in the larvae of *Bombus terrestris* (Collins et al. 2017). Exogenous microRNAs found in bee food, with worker jelly having a higher abundance, may also play a role in caste development, as seen with *ame-miR-184* that was identified as important for regulating caste morphology (Guo et al. 2013). Additionally, a study found similar patterns of microRNA expression among queens and workers with activated ovaries compared to virgin queens and workers with inactivated ovaries (Macedo et al. 2016). When queen ovaries become active, microRNAs, such as *miR-14*, play a crucial role in regulating egg-laying as their inhibition resulted in higher egg production through targeting the mRNA of the ecdysone receptor in honey bees (Chen and Fu 2021).

MicroRNAs exhibit nuanced regulation by partially suppressing gene expression to prevent overexpression (Posadas and Carthew 2014). Several microRNAs are specifically involved in the regulation of royal jelly-related processes. For instance, there is a positive correlation among microRNAs targeting genes upregulated in response to royal jelly ingestion, suggesting their presence to curtail the overexpression of these genes and prevent deleterious effects (Guo et al. 2013). Additionally, specific microRNAs have been linked to the production of royal jelly by nurse bees, while others are associated with honey processing in foragers, indicating their role in regulating glandular functions associated with different tasks (Shi et al. 2021). Beyond royal jelly regulation, microRNAs also play critical roles in worker polyethism within social insect societies. Changes in miRNA expression have been observed in the brains of foraging workers compared to hive workers (Liu et al. 2012). For example, one study found specific brain microRNAs associated with young nurses and others upregulated in older foragers, highlighting their importance in age-related behavioural changes (Behura and Whitfield 2010). Notably, the most abundant microRNA found in brains of honey bees, *ame-miR-2796*, is known to be important for regulating forager behaviour and was found to be conserved among several social insects (Greenberg et al. 2012). Additionally, microRNAs exhibit differential expression among nurses, foragers, and newly emerged bees within the hypopharyngeal glands, which undergo alterations during development, implicating these microRNAs in developmental processes (Shi et al. 2021). Taken together, the diverse functions and caste-specific expression patterns of microRNAs support their role in the evolution of social complexity, providing regulatory flexibility that enables phenotypic divergence without requiring large-scale genomic changes.

### Circular RNA

Circular RNAs (circRNAs) have garnered significant attention in recent years due to their unique structure and regulatory functions. Characterized by a closed-loop structure, circRNAs possess increased stability and resistance to RNAse degradation compared to linear RNAs, making them attractive candidates as stable biomarkers capable of accumulating in biological systems (Zhou et al. 2020). Initially considered as splicing by-products, circRNAs have emerged as important regulators of gene expression, particularly in post-transcriptional regulation, including as competing endogenous RNA (ceRNA), which are microRNAs that regulate other types of RNAs and modulates mRNA availability (Hansen et al. 2013; Memczak et al. 2013; Salzman et al. 2013; Ashwal-Fluss et al. 2014; Ala 2020). These circRNAs can act as sponges and bind to other microRNAs or RNA binding proteins in an effort to block their effects (Panda 2018). CircRNAs play diverse roles in various aspects of bee biology, including social behaviour, task specialization (Tholken et al. 2019), reproductive division of labour (Chen et al. 2019) and development (Chen et al. 2020). For instance, Tholken et al. (2019) identified correlations between circRNAs, DNA methylation, and task allocation in bees. They found circRNAs enriched for annotations related to memory formation and neuromuscular synaptic transmission regulation, which are crucial for tasks such as foraging and hive maintenance (Tholken et al. 2019). Furthermore, a specific circRNA, *ame_circ_0001780* (*circAmrad*), was correlated with the transition from nurse to forager bees, and another *circAmrsmep2* was found to increase with worker age, indicating their involvement in behavioural plasticity and response to environmental cues (Tholken et al. 2019). These findings suggest that circRNAs may have contributed to the evolution of eusociality by enabling flexible gene regulation necessary for task specialization and the emergence of division of labour among castes. CircRNAs have also been implicated in reproductive processes in bees. Chen et al. (2019) identified the circRNA *ame_circ_0010349* as upregulated in mating queens, suggesting its role in ovary activation and reproductive division of labour. Furthermore, Chen and Shi (2020) identified circRNAs differentially expressed among workers and queens, suggesting their involvement in the reproductive division of labour by potentially interacting with microRNAs in ecdysone and vitellogenin pathways. Such caste-specific regulatory interactions point to circRNAs as potential contributors to the evolutionary stabilization of queen and worker roles within a eusocial system.

Additionally, Chen et al. (2023) identified a circular RNA, *ame_circ_0002015*, that influences ovary activation in *Apis mellifera*. Experimental manipulation revealed a positive correlation between its expression and the number of eggs laid by queens, linking circRNA activity to reproductive function, which is an essential caste-specific trait (Chen et al. 2023). Interestingly, *ame_circ_0002015* was also found to be regulated by *miR-14-3p*, highlighting a complex regulatory network between circRNAs and microRNAs that may fine-tune oviposition (Chen et al. 2023). Beyond reproduction, circRNAs also play stage-specific roles in larval development in bees. Chen et al. (2020) identified circRNAs participating in midgut development, with differential expression patterns suggesting stage-specific roles in midgut maturation. These findings provide insights into the molecular mechanisms underlying organ development and tissue specialization in bees. Recently, Zhang et al. (2023) conducted a study focusing on circRNAs within the gut of developing larvae of *Apis mellifera ligustica*. They found significant differential expression among larvae of different ages where circRNAs that were differentially expressed were involved in growth, development, regulation, and metabolism (Zhang et al. 2023). Notably, they identified a new circRNAs, *Novel_circ_000838*, that interacts with the target microRNA *miR-6000a-3p*, providing evidence for a ceRNA networks involved in regulating microRNAs during larval development (Zhang et al. 2023). These findings underscore the role of circRNAs in regulating key developmental and reproductive processes. Since such traits are central to caste differentiation in eusocial insects, dynamic RNA-based regulatory networks, especially involving circRNAs and microRNAs, may have played a pivotal role in the evolution of social complexity in bees. By modulating gene expression without altering coding sequences, circRNAs provide a flexible mechanism through which developmental pathways, and thus caste-specific traits like morphology, physiology, and behaviour, can evolve and diversify.

Circular RNAs have also emerged as critical regulators of immunity in bees. For example, one study examined the effects of the neonicotinoid insecticide Dinotefuran on circRNA expression in honey bees, revealing an increase in differentially expressed circRNAs in honey bee brains over time, particularly those targeting genes involved in immune pathways (Huang et al. 2022). Studies of *Apis mellifera ligustica* exposed to either *Ascosphaera apis* (Ye et al. 2022) or *Vairimorpha ceranae* (Chen et al. 2022a) have highlighted the involvement of circRNAs in immune-related pathways at different ages and stages of infection. In a study on *Apis cerana* infected with *Nosema ceranae*, researchers identified novel circular RNAs implicated in immunity, along with ceRNAs that sequester microRNAs associated with various aspects of the immune pathway (Zhu et al. 2022). Notably, they discovered that one of the newly identified circRNA genes was found in the Hippo signaling pathway, which is known to be involved in regulating cell proliferation and growth and the regeneration of organs (Tang et al. 2022). This finding suggests that differentially expressed circRNAs can act as markers of damage in the midgut due to *Nosema* infection. Consequently, the host (worker) may utilize these circRNAs to regulate transcription, facilitate cellular renewal and modulate their immune response (Zhu et al. 2022). As circRNA-mediated regulation can influence how bees respond to pathogens and environmental stressors, these mechanisms may play a role in shaping adaptive responses over time. Understanding the roles of circRNAs in bees not only provides insights into their complex biology but also offers opportunities for developing strategies to mitigate threats to bee health and conservation. Taken together, the diverse functions of circRNAs in development, behaviour, and immunity suggest they are not only important for individual plasticity and defense, but also represent molecular innovations that may have facilitated the emergence and refinement of caste-specific traits, which are key steps in the evolution of eusocial complexity in bees.

## Comparative Genomic Studies Uncover Features of Evolution

Comparative genomic studies offer a robust framework for understanding the evolution of eusociality by examining genomic differences among species with varying social behaviour. For instance, a study using whole genome sequencing on *Lasiogossum albipes*, a species with both social and solitary populations, identified single nucleotide polymorphisms and gene expression patterns that are associated with variation in social behaviour (Kocher et al. 2018). They identified the gene *syntaxin 1a*, a gene involved in neurotransmission, as highly genetically differentiated between social and solitary *L. albipes* and showed behaviour-specific gene expression (Kocher et al. 2018). Analyses within conspecific populations exhibiting polymorphic behaviour provide inherently controlled comparisons, which reveal aspects of genome evolution related to social transitions. For example, comparative studies have identified gene regulation to be particularly important for the evolution of eusociality, as large-scale studies across multiple species have identified it as a key mechanism underlying the evolution of social behaviour (Wittwer et al. 2017; Kocher et al. 2018; Rehan et al. 2018; Kapheim et al. 2020a)(Kapheim et al. 2015; Thompson and Richards 2016). In a study analyzing 10 bee species with diverse origins of sociality, Kapheim et al. (2015) found that social behaviour correlates with enhanced gene regulatory capacity, evidenced by increased activity of transcription factors and a higher number of transcription factor binding sites. Similarly, studies on conserved Non-Coding Alignable Regions (NCARs) of DNA among 11 species from various origins and levels of sociality suggested that gene regulation is important for eusocial genome evolution (Rubin et al. 2019). Specifically, these regulatory changes are associated with evolutionary rate shifts in non-coding regions that are linked to social behaviour and divisions of labour. Furthermore, a recent study comparing 17 different species to identify genomic features associated with the transition between solitary and social behaviour found that in lineages where sociality was lost there were relaxed selective pressures on taxonomically restricted genes and gene regulatory elements suggesting that the transition to social behaviour requires regulation of gene expression and selection of genes that promote enhanced regulation (Jones et al. 2023).

Through comparative genomics studies, researchers have gained insights into the distinct patterns of adaptive molecular evolution among various bee species, reflecting their diverse social behaviours. For instance, in a comparative analysis between three *Bombus* species and two *Apis* species, Harpur et al. (2017) revealed signs of adaptive divergence with evidence of strong positive selection found among genes associated with cognition and sensory perception in honey bees and in metabolic genes in bumble bees. Moreover, when comparing the selection coefficients of genes upregulated in queen relative to workers (queen-biased genes) and genes upregulated in workers relative to queens (worker-biased genes) in each species, the study found higher selection for queens-biased genes in bumblebees, while the opposite trend was observed in *Apis* species, suggesting distinct mechanisms of adaptive evolution (Harpur et al. 2017). A comparative genomics study investigated adaptive evolution in honey bees, bumblebees, and paper wasps, hypothesizing parallel patterns of adaptive evolution among species with similar social structures (Dogantzis et al. 2018). Consistent with previous research, it found higher adaptive evolution in genes biased towards reproductive individuals in bumblebees and paper wasps compared to worker-biased genes among honey bees (Dogantzis et al. 2018). The study also observed a greater overlap of positively selected genes between primitively eusocial species that exhibit cooperative brood care and reproductive division of labour but without strong morphological differences between castes (bumblebees and paper wasps), relative to advanced eusocial honey bees, suggesting convergent adaptive molecular evolution (Dogantzis et al. 2018). Another study leveraged comparative genomics and analyzed several bumblebee species with distinct behaviour including the non-social cuckoo bumblebee (subgenus *Psithyrus*) that act as social parasites (Fouks and Lattorff 2016). This study found varying patterns of selection in targeted genes known to be involved in social behaviour as well as their regulation among species exhibiting social versus parasitic behaviour, highlighting the utility of social parasites in studying mechanisms of selection within a comparative genomic framework (Fouks and Lattorff 2016). Overall, comparative genomic studies illuminate the dynamic interplay between genomics and behaviour, offering a comprehensive understanding of the intricate mechanisms driving the emergence and diversification of sociality in bees.

## Ecology

### Museomics

Museum collections are a valuable source of historic DNA (Bi et al. 2013), whose sequence information can facilitate genomic investigations of adaptation across a wide variety of insects including bees (Cavill et al. 2022; Cilia et al. 2022), moths (Call et al. 2021), locusts (Goodman et al. 2023), beetles (Toussaint et al. 2021; Cohen et al. 2022), butterflies (Grewe et al. 2021), ants (Eguchi et al. 2020), flies (Shpak et al. 2023; Strunov et al. 2023), and others (Schmitt et al. 2018; Derkarabetian et al. 2019; Korlevic et al. 2021). While invaluable for conservation genomics, these data typically reflect very recent evolutionary trajectories on the order of fewer than 100 to 200 generations and therefore offer unique insight into contemporary responses to anthropogenic pressures. Beyond genetic material, pollen can be extracted from both modern and preserved specimens to identify the changes in floral resource composition, assessing landscape changes, and analyzing plant-pollinator interactions over longer timeframes using tools such as pollen metabarcoding (Bell et al. 2023; Wizenberg et al. 2023a , 2023b). Overall, museum samples play an important role in uncovering patterns of genetic adaptation and ecological responses to environmental change across insect taxa.

Studies of wild bee species have highlighted the power of museum collections for tracking evolutionary and ecological shifts. For example, by comparing the genomes of the small Carpenter bees *Ceratina calcarata* and *Ceratina dupla* from contemporary populations to those sampled 50 years prior, Brasil et al. (2023) identified a reduction in genetic diversity and effective population size in this relatively short time frame. These species also show population genetic evidence that proposes a potential adaptation to climate change and insecticide use (Brasil et al. 2023), although experimental confirmation of this has yet to be demonstrated. Similarly, a study in *Ceratina* found that with agricultural expansion, male body size decreased while female body size increased, suggesting distinct selective pressures (Kelemen and Rehan 2021; Pope et al. 2023). Additionally, studies have highlighted decreases in genetic diversity in at-risk species like *Bombus occidentalis*, suggesting reduced genetic resilience to environmental changes or disease resistance (Rohde et al. 2024). Pollen metabarcoding has expanded this type of work as seen in a study conducted in Cuxhaven, Germany, that analyzed pollen collected from contemporary bumblebees and compared them with historic specimens from 1968 to 1969, revealing notable changes in foraging behaviour, pollination networks and community composition (Kolter et al. 2023). Similarly, Simanonok et al. (2021) applied pollen metabarcoding to modern and museum samples of the endangered rusty patch bumblebee (*Bombus affinis*) spanning a century (1913 to 2013). Their findings indicated no significant differences in floral resource usage over time, suggesting that regional variations, rather than floral availability, may be unrelated to the species' decline (Simanonok et al. 2021). In another study, Gous et al. (2021) compared pollen metabarcoding data from modern leaf-cutter bees (Megachilidae) in South Africa with museum specimens and discovered that bees with a wider foraging range exhibited a broader spectrum of floral resource use, but noted no significant temporal differences (Gous et al. 2021). Taken together, this demonstrates how historical DNA and pollen samples from wild bee specimens can shed light on recent demographic, adaptive or ecological dynamics.

In *Apis mellifera*, museum-based studies have offered detailed insights into population history and management. A study of California honey bees spanning 105 years revealed genetic changes over time, including the introgression of African ancestry and alleles into managed populations (Cridland et al. 2018). In a study of Swiss honey bee museum specimens (1879 to 1959), Parejo et al. (2020) found higher genetic diversity among modern samples compared to historical, museum samples, suggesting a lack of population bottleneck potentially due to beekeeping practices. However, this analysis did not account for the strong population structure and lineage turnover in European honey bees (e.g. from the native M lineage to C or admixed individuals). More recent studies that incorporate lineage distinctions, such as Espregueira Themudo et al. (2020) who also analyzed museum specimens and Leroy et al. (2024) who inferred demographic history from present-day linkage disequilibrium patterns, have instead reported signatures of population bottlenecks. These findings highlight the complexity of honey bee demographic history and the importance of considering population structure in such analyses.

Museomics, however, remains subject to various forms of bias that limit the reliability of its outcomes. For instance, researchers can only compare results against what has been preserved, leading to observation bias—comparisons are inherently limited to the available reference data (Shaffer et al. 2025). Additionally, DNA amplification methods, including the choice of Taq polymerase and the composition of primers, can introduce amplification bias (Silverman et al. 2021; van der Loos and Nijland 2021). Since species are typically analyzed as parts of a whole community, under-detection of one species will, by definition, inflate the apparent abundance of others within that group (McLaren et al. 2019). Future studies should prioritize minimizing these sources of bias by expanding reference databases through additional taxonomic sampling efforts to reduce observation bias. By improving the design of primers to either be universal or taxon-specific, researchers can overcome the limitation of poorly designed primers that may be binding to multiple species and increasing the abundance of one species artificially, thus limiting amplification bias. Another suggestion is to use a reference species to calibrate the metabarcoding data and thus be able to use absolute DNA quantities instead of relative proportions, although this method is dependent on exact primer matches (Shaffer et al. 2025). Addressing these challenges through methodological improvements and careful calibration will be essential to enhance the accuracy and reliability of metabarcoding as a tool for biodiversity assessment.

### Environmental ‘Omics

Environmental DNA (eDNA) is DNA that is collected from diverse environmental samples, such as water (Miyata et al. 2022), air (Klepke et al. 2022; Roger et al. 2022; Garrett et al. 2023), spiderwebs (Gregoric et al. 2022), and even honey (Bovo et al. 2018, 2020; Pathiraja et al. 2023). eDNA has also been widely used in microbiome analysis within ecological contexts. For more information, we refer readers to several reviews (Ladin et al. 2021; Patin and Goodwin 2022; Cook et al. 2025), as we focus on how eDNA and metagenomics reveal insights into environmental genomics. eDNA can be used to estimate species richness and construct interaction networks within ecosystems, such as detecting pollinator interactions within the environment without needing direct observation. For example, eDNA analysis can be coupled with advanced sequencing techniques like next-generation sequencing (e.g. Ion Torrent) for shotgun metagenomic analysis of honey eDNA (Bovo et al. 2018) or used in conjunction with metabarcoding (Harper et al. 2023) to understand the environmental dynamics of bee habitats and their interaction networks with plants (Newton et al. 2023), animals (Boardman et al. 2024), and pathogens or viruses (Bovo et al. 2020) (Paluoja et al. 2025). These approaches can help reveal how environmental pressures influence bee behaviour, pathogen resistance, and plant-pollinator co-evolution, providing indirect evidence of selective forces shaping bee genetic diversity and adaptive traits over time. For example, long-term eDNA and metabarcoding monitoring such as analyses of bee-collected pollen or environmental samples from herbarium specimens, can track shifts in plant-pollinator community composition and phenology, offering valuable insights into evolutionary responses to climate change, land use, and pathogen dynamics (Waters et al. 2025; Wizenberg et al. 2025). Additionally, eDNA has demonstrated practical utility for detecting bee-associated pests and pathogens (Ribani et al. 2020, 2022) and even rare or cryptic plant species visited by bees (Batchelor et al. 2023), offering tools for long-term monitoring of ecological and evolutionary responses. There are several limitations that need to be considered with eDNA methods including that eDNA degrades over time due to temperature, UV light or distance which can limit its detection (Ruppert et al. 2019). Furthermore, eDNA is not currently able to accurately detect biomass, which is the aggregate mass of individuals of a certain species or group, based on concentrations of DNA detected (Danziger et al. 2022). These collected samples also lack contextual information, such as the age and life-stage of the individuals contributing to the pooled sample, which is an inherent limitation when gathering DNA shed by the organism (Couton et al. 2023). Despite these limitations, eDNA remains a powerful and versatile tool that can complement other molecular techniques and can serve as an efficient starting point for identifying the presence and diversity of organisms in an environment and guiding more targeted ecological and evolutionary studies.

### Pollen Metabarcoding

**Fig. 3.**
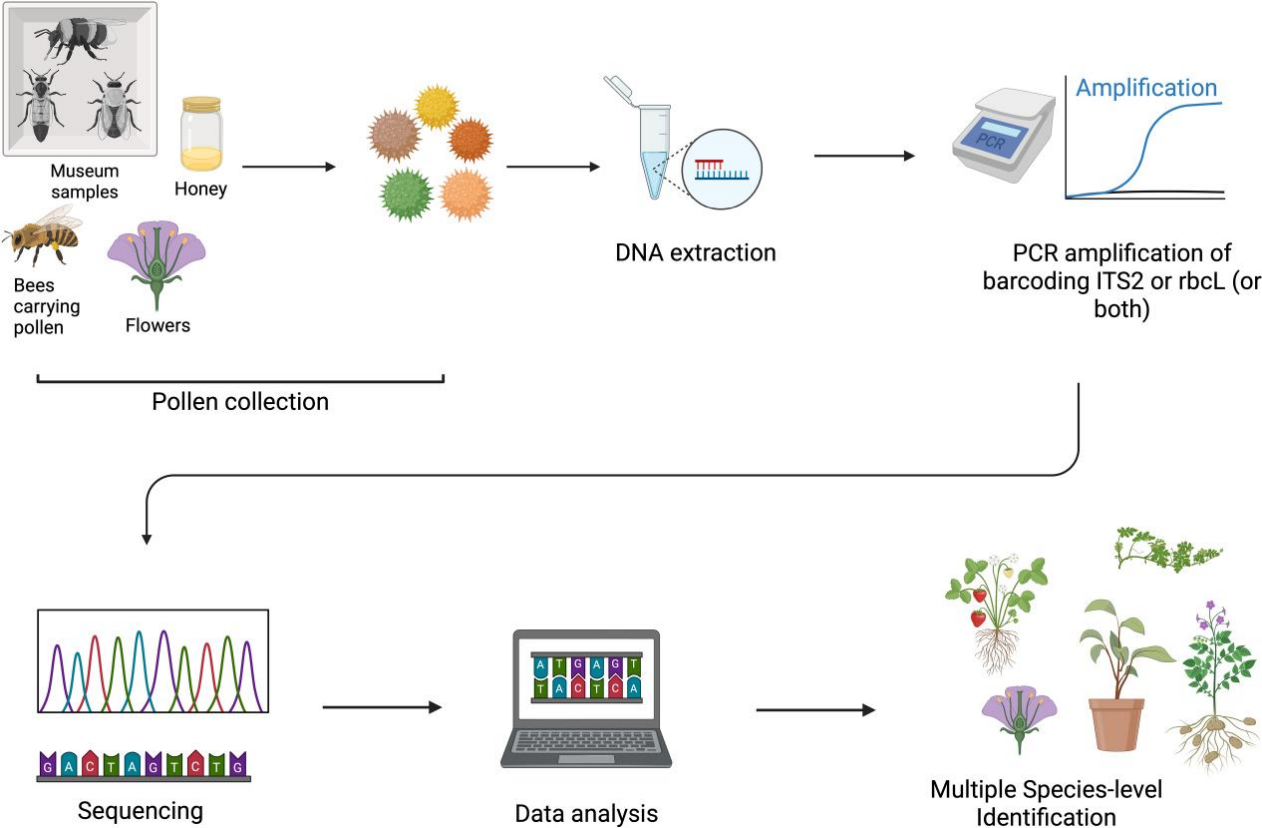
Schematic overview of a pollen metabarcoding workflow for plant identification. Pollen is collected from diverse sources such as bees, flowers, museum specimens, or honey. DNA is extracted from mixed pollen samples, followed by PCR amplification targeting standard plant barcode regions (e.g. ITS2 or rbcL, or both). Amplified products are sequenced and resulting reads are analyzed bioinformatically to identify plant taxa at the species level. This approach enables detailed reconstruction of plant-pollinator interactions and floral resource use.

Pollen metabarcoding provides crucial insights into the complex and often elusive network of bee interactions (Wizenberg et al. 2023a, 2024). Given the wide variety of plants that bees interact with, pollen metabarcoding offers a window into what plant species bees are visiting at different times of the year, enhancing our understanding of their intricate ecological relationships. Recent advances in wet lab and bioinformatic protocols have demonstrated that pollen metabarcoding can quantify the types and relative amounts of pollen collected by bees as accurately as traditional (i.e. taxonomy-based) methods, while requiring considerably less time and cost (Fig. 3). A recent study utilizing these new methods identified that honey bees visit a much greater diversity of non-flowering plants than previous anticipated (Wizenberg et al. 2023b). For example, genetic material from sporophytes, bryophytes as well as different species of green algae were all detected in honey bee beebread (i.e. stored pollen provisions) (Wizenberg et al. 2023b). While the relevance of such visits is not fully understood, it suggests that honey bees and likely other bee species may be important dispersal vectors for non-flowering plants as well as flowering plants (Wizenberg et al. 2023b). Analysis of bee bread collected from a very large cohort of honey bee colonies from across Canada discovered that honey bee foraging preferences were strongly associated with dichotomous patterns of stressor exposure (Wizenberg et al. 2024). This pattern showed that colonies with low dietary diversity tended to be exposed to harmful agrochemicals, while colonies with high dietary diversity where more likely to be exposed to pathogens (Wizenberg et al. 2024). The ability of social bees to “sample” pollen from a variety of plants over the year makes them particularly suited to monitor changes in plant phenology over time. In a small pilot study in the city of Toronto (Canada), Wizenberg et al. (2025) discovered that pollen sampled from honey bee colonies was able to predict the onset of flowering in a large number of plants. This finding opens the door to characterizing changes in plant phenology over time by sequencing pollen collected by bees. Pollen metabarcoding is therefore emerging as a powerful tool to elucidate landscape dynamics and plant-pollinator interactions across different temporal scales.

While these studies show a promising new direction that researchers may take to understand the behaviour of bees, plant flowering, and other ecological relationships among plants and animals, it's important to consider the biases that are still inherently present in this technology. Researchers must often use a combination of pollen metabarcoding loci such as both ITS2 and *rbcL* to be able to capture different taxonomic groups but even those are limited by the contents of their libraries (Arstingstall et al. 2021). Furthermore, the reference databases are also a limiting factor in the robustness of these studies as they too are often limited to a specific geographic location or may only include certain native plants (Milla et al. 2022; San Martin et al. 2024). Thus, important ecological features are lost among these studies due to a lack of robust libraries and databases. Future studies should therefore aim to expand these barcode libraries so studies can better capture the accurate interactions among plants and animals.

## Conclusion

The use of genomic tools, methods and techniques have revolutionized our understanding of bee phylogeny, offering new insights into the evolutionary relationships and diversification of this highly varied group. Advances in next-generation sequencing, the application of ultraconserved elements (UCEs), and phylogenomic approaches have refined long-standing questions about bee evolution, enabling the reconstruction of more accurate gene and species trees. These tools have provided deeper resolution of bee lineages across families and subfamilies, revealing key evolutionary events such as the origins of cleptoparasitism, which is a form of parasitism where an insect will lay their eggs in the nest of another species and will use the host's resources to provision for their young (Cardinal et al. 2010) and the diversification of major clades like Apinae and Eucerinae. UCE-based studies have clarified taxonomic ambiguities, resolved phylogenetic positions within complex lineages, and reshaped our understanding of the timing and geographic origins of various bee groups. The expanding genomic datasets, including those derived from museum specimens and environmental DNA, are continuing to uncover both historical and contemporary patterns of bee evolution, offering powerful methods for tracking ecological shifts and genetic changes over time. These findings underscore the importance of phylogenomics on the study of bee evolution, with future research poised to explore even broader taxonomic scales and ecological contexts.

We also highlight the growing understanding of the molecular mechanisms that underpin eusocial behaviour in bees, focusing on the roles of alternative splicing, long non-coding RNA (lncRNA), microRNA (miRNA), circular RNA (circRNA), and methylomics in shaping social phenotypes. Through advances in genomics and transcriptomics, researchers have identified key regulatory pathways that influence division of labour, reproductive roles, foraging behaviour, and immune responses within bee colonies. While alternative splicing generates diversity in caste-specific behaviours, lncRNAs and microRNAs contribute to gene regulation that is critical for development, social interaction, and immune function. CircRNAs, with their stability and regulatory potential, are emerging as important factors in behaviour and immunity. The findings reviewed underscore the complex molecular toolkit that supports social evolution in bees, but they also point to the need for further research to fully understand the interplay between these mechanisms and their roles in different social contexts. This growing body of work not only advances sociobiological theory but also offers potential insights for conservation efforts and understanding environmental impacts on bee populations.

Together, advances in phylogenomics and molecular biology have greatly expanded our understanding of bee evolution and behaviour. By combining tools that help resolve evolutionary relationships with those that uncovered the molecular basis of social traits, we are starting to build a more complete picture of how bees have diversified and adapted to different environments. This growing body of work not only sheds light on the mechanisms behind social evolution and lineage diversification, but also provides useful context for conservation, especially as bees face increasing environmental pressures. As genomic datasets continue to grow and become more accessible, future studies will be well-positioned to link broad evolutionary patterns with fine-scale molecular processes and improve our ability to understand and predict how bees may respond to changing conditions.

## Data Availability

No new data were generated or analysed in support of this research.
